# Features Suggestive of Coexisting Amyotrophic Lateral Sclerosis in Patients With Spinal Stenosis and Influence of Spinal Decompression

**DOI:** 10.7759/cureus.51587

**Published:** 2024-01-03

**Authors:** Jeremy Hill, Nirav Sanghani, Yuebing Li

**Affiliations:** 1 Department of Neurology, Neuromuscular Center, Cleveland Clinic, Cleveland, USA

**Keywords:** rate of progression, electromyography, surgery, amyotrophic lateral sclerosis, spine stenosis

## Abstract

Background: Spinal stenosis and amyotrophic lateral sclerosis (ALS) can co-occur and both manifest as signs of dysfunction of lower and/or upper motor neurons. Few studies have identified factors that alert the diagnosis of ALS in patients with spinal stenosis, and the influence of spinal decompression surgery on ALS progression remains unclear.

Objective: The objective of this study is to describe factors that are suggestive of an ALS diagnosis in patients with spinal stenosis and influence of spinal decompression surgery on the progression of ALS

Materials and methods: A retrospective review of the institutional ALS database and electronic medical records was performed to identify patients with coexisting diagnoses of ALS and moderate to severe cervical and/or lumbosacral spine stenosis. Identified patients were divided into two subgroups: those with spinal decompression surgery and those without. Comparisons of clinical features and progression of ALS were made between subgroups.

Results: A total of 77 patients with ALS and coexisting moderate to severe cervical or lumbosacral spine stenosis were included. Among them, 50 patients underwent spinal decompression surgery and 27 did not. In comparison to patients with spinal decompression, patients without spinal decompression surgery were seen more frequently by neurologists (74% versus 26%), had less prominent radicular pain (19% versus 50%), demonstrated more frequent bulbar signs (30% versus 8%), experienced more likely weight loss (41% versus 4%), and disclosed more noticeable axonal loss changes on electromyography. Spinal decompression surgery did not modify the progression of ALS based on ALSFRS-R score change and analysis of survival duration.

Conclusion: Our study identified a number of useful features that are suggestive of an ALS diagnosis when evaluating patients with spinal stenosis and may support the performance of spinal decompression surgery in a subset of selected ALS patients with symptomatic spinal stenosis.

## Introduction

Spinal stenosis occurs frequently in patients with amyotrophic lateral sclerosis (ALS) [1-3]. Spinal stenosis could result in radiculopathy and/or myelopathy, manifesting as signs of dysfunction of lower motor neurons, upper motor neurons, or both [1]. Thus, ALS may first be misdiagnosed as spinal stenosis, and ALS patients could undergo unnecessary spinal decompression surgery [4-7].

In patients with a putative or confirmed diagnosis of ALS, conflicting data exist on whether spinal decompression surgery could alter ALS progression [8,9]. For example, Pinto et al. noted that in a small group of ALS patients, disease progression accelerated during a three-month period immediately following a variety of surgeries including spinal decompression. Authors speculated that anesthetic and/or surgical stress might have hastened the underlying neurodegeneration [8]. Alternatively, Yoshor et al. observed that spinal decompression surgery did not modify the ALS progression rate as reflected by a change in the Appel ALS total score and the overall ALS patient survival [9]. Reasons to account for differences among the above studies remain unclear, but possibly related to variations in the ALS stage, ALS progression speed, patient age, and differences in surgical procedures [8].

This study intended to identify a group of ALS patients with coexisting spinal stenosis and divide the patients based on the performance of spinal decompression surgery. By comparing those ALS patients who underwent spinal decompression surgery and those who did not, we aimed to answer the following two questions: 1. Are there clinical features in a patient’s presentation that may signal that ALS rather than spinal stenosis is the driving factor of their clinical manifestation? 2. Does spinal decompression surgery alter the progression of ALS?

## Materials and methods

This retrospective case-control study was conducted in our tertiary referral center (Neuromuscular Center, Cleveland Clinic, Cleveland) and was approved by our institutional review board. Patients included were those who fit the following two criteria: (1) presented with neurologic deficits and were found to have moderate to severe cervical or lumbosacral central canal or foraminal spinal stenosis on neuroimaging that may explain at least part of the initial presentation between 2005 and 2020 and (2) were later diagnosed with ALS by the Cleveland Clinic Neuromuscular Center as the primary etiology of their symptoms/signs during the same disease course. The initial list of patients was identified via one of the following two methods: (1) a search of the institutional electronic medical record using the following international classification of diseases (ICD)9 and ICD10 codes: 335.20, G12.21, 723.0, 724.02, M48.07 and M48.02 and (2) a review of each patient within our institutional ALS patient database for the presence of cervical or lumbosacral spinal stenosis diagnoses. The electronic medical record of each patient was retrospectively reviewed, and only those who fit the above criteria were included. Patients with mild central canal or foraminal stenosis or patients with incomplete evaluation were excluded.

Data collection included demographics, provider specialty, initial symptoms, presence or absence of neuropathic pain, weight loss at initial presentation, onset date and region, neurologic exam features, presence or absence of bulbar signs (e.g., dysarthria, dysphagia, sialorrhea, tongue atrophy and fasciculations), initial diagnoses, electromyography (EMG) results before and after surgery as available, MRI results, interval duration from symptomatic onset to ALS diagnosis, the Revised Amyotrophic Lateral Sclerosis Functional Rating Scale (ALSFRS) scores if available, and operative notes. The initial EMG reports were reviewed, and an average number of root/level involvement was calculated based on the presence of active and/or chronic axonal loss changes demonstrated on needle exam per cervical or lumbosacral segment. EMG findings of active axonal loss changes included the presence of fibrillation potentials and positive sharp waves, whereas signs of chronic axonal loss changes consisted of long-duration motor unit potentials (MUPs), reduced MUP recruitment with increased rate of firing, and MUP instability.

Results were given as means, standard deviations, ranges for continuous variables, and percentages and counts for categorical variables. Group comparison was achieved via two-sample Mann-Whitney U tests. Kaplan-Meier survival analysis was used to compare survival data. The p-value <0.05 was considered as statistically significant.

## Results

A total of 1,147 ALS patients were initially screened for spinal stenoses. Among them, 77 patients had moderate to severe spine stenosis based on radiological studies and were included in the study. Table 1 lists demographic information for all included patients. 

**Table 1:** Patient demographics

Features	Number
Total number of patients	77
Mean age at symptom onset, years	63.3
Age range at symptom onset, years	33 to 84
Race, Caucasian (%)	63 (82)
Race, African American (%)	13 (17)
Race, unknown (%)	1 (1)

In 76 patients, onset was in the limb (cervical or lumbosacral) region, and the other patient had a craniobulbar onset. Sixty-one (79.2%) patients were evaluated by surgery for consideration of spinal decompression. Among them, 50 patients underwent cervical (n=23), lumbar (n=25) or both (n=2) spinal decompression surgeries. A total of 27 ALS patients with cervical (n=20) or lumbar (n=7) spinal stenosis did not undergo surgery. In all patients, spinal stenosis occurred in the cervical or lumbar segments that were congruent with the onset limb. Among ALS patients who underwent surgery, 65% underwent EMG testing prior to surgery, and all patients received EMG prior to their ALS diagnosis. ALS was mentioned in the differential diagnosis for 12 (24%) of 50 patients in the surgery group prior to their spinal decompression surgery. Patient demographic information is listed in Table 1.

Table 2 shows a comparison of the clinical and electrophysiological features between the groups of ALS patients with and without spinal decompression surgeries.

**Table 2:** A comparison of clinical features between patients with and without spinal decompression surgery p-value <0.05 was considered as statistically significant. ALS, amyotrophic lateral sclerosis; SD, standard deviation; EMG, electromyography; ALSFRS, amyotrophic lateral sclerosis functional rating score

Feature	ALS patients without surgery (N=27)	ALS patients with surgery (N=50)	p-value
Female sex, N (%)	7 (26)	17 (34)	0.47
Onset age (mean± SD), years	64.7± 12.0	62.5 ± 10.8	0.48
Neurology as initial provider seen, N (%)	20 (74)	13 (26)	0.006
Prominent pain at presentation, N (%)	5 (19)	25 (50)	0.007
Upper motor neuron signs at presentation, N (%)	21 (78)	26 (52)	0.08
Bulbar signs at presentation, N (%)	8 (30)	4 (8)	0.01
On-going weight loss at presentation, N (%)	11 (41)	2 (4)	0.0002
Number of affected levels per segment (cervical or lumbar) on EMG (mean± SD)	4.6 ± 0.9	3.2 ± 1.4	0.0004
Time to diagnosis of ALS from onset (mean± SD, month)	19.4 ± 19.4	19.6 ±17.5	0.48
Change in the ALSFRS score per month (mean± SD)	-0.94 ± -1.7	-0.77 ± -0.95	0.69

**Figure 1:**
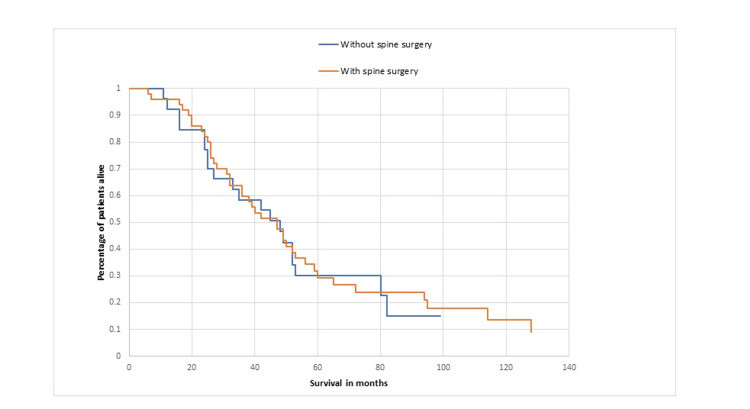
A comparison of survival in ALS patients with and without spinal decompression surgery ALS, amyotrophic lateral sclerosis

No significant differences were observed regarding age, sex, frequency of upper motor neuron signs, and interval duration from symptomatic onset to ALS diagnosis. Patients without surgery were more frequently seen by a neurologist rather than a neurosurgeon or orthopedic surgeon at the first visit, had less prominent radicular pain on presentation, demonstrated bulbar signs on neurologic exam, experienced weight loss, and disclosed more noticeable axonal loss changes in EMG based on the number of involved root levels per cervical/lumbosacral segment. Post-spinal decompression surgery, weakness improved transiently in 7 (14%) of ALS patients while radicular pain improved in 20 (40%). Surgery did not significantly impact the ALSFRS-R score rate change (Table 2). Kaplan-Meier analysis showed no difference in survival between patients in the spinal decompression surgery and no-surgery subgroups (Figure 1), with the median survival duration of 40 months for the subgroup of patients with surgery and 42 months for the subgroup without (p= 0.64). 

## Discussion

Spinal stenosis is often considered in the differential diagnoses of ALS [1,3,10]. Richards et al. reviewed various studies describing the rate of misdiagnosis in ALS and reported that cervical myelopathy, disc herniation, and cervical and lumbosacral radiculopathy were among the frequently occurring misdiagnoses in the evaluation of ALS [10]. Importantly, misdiagnosed patients more likely underwent unnecessary surgeries [5,6,10,11]. Yoshor et al. previously reported that 4.2% of unrecognized ALS patients underwent spinal decompression surgery [9]. Similarly, 50 (4.4%) ALS patients in the current study received similar surgeries. Yoshor et al. noted a delay of 13 months in making an ALS diagnosis due to spine decompression [9]. We did not observe a significant difference in the interval duration between symptomatic onset and establishment of the ALS diagnosis between subgroups of patients with and without surgery, but we cannot exclude the possibility that the group of ALS patients who underwent spinal decompression surgery could have been diagnosed sooner had they not undergone surgical decompression.

In patients with ALS and coexisting spine disease involving the same segment, symptoms or signs may not be clearly attributed to ALS or spine disease [1,3,5,6,8,9]. Our study identified several features that were significantly different between patients who had spinal decompression surgery and those who did not. More ALS patients in the surgery group were initially evaluated by a surgeon rather than a neurologist. This is similar to previous studies reporting that ALS misdiagnosis was more likely in those seen by non-neurologists [10,11]. In our study, patients who received spinal decompression surgery had a lower incidence of craniobulbar symptoms, similar to the finding reported by Yoshor et al. [9]. Possible reasons include that patients with limb-onset are less likely to see a neurologist and have a longer list of differential diagnoses than those of bulbar onset [10]. In addition, several features such as the presence of radicular pain, lack of weight loss, and less extensive axonal loss changes on EMG made it more likely for ALS patients to receive spinal decompression surgery (Table 2). Therefore, paying particular attention to these features could help avoid unnecessary spinal decompression surgery in ALS patients. Ancillary studies such as evoked potentials by transcranial magnetic stimulation, diffusion tens imaging, and measurement of neuron-specific enolase level could also be helpful in distinguishing ALS from degenerative spinal disorders [12-16].

Several previous studies suggest that surgical intervention can be associated with an accelerated progression of ALS or decreased survival [5,8,9,17]. Our study was consistent with those of Yoshor et al., not revealing a significant difference in the survival or progression of ALS based on examinations of ALSFRS-R score change and Kaplan-Meier survival analysis [9]. In selected ALS patients, there may still be indications for spinal decompression surgery [1,5,9]. Our study found that 40% of ALS patients who underwent spinal decompression surgery reported some improvement in their neuropathic pain. In addition, in some cases it is clear that spinal stenosis serves as the main driving force of clinical manifestations. Under such circumstances, spinal decompression surgery may be warranted, especially in those patients with a rapid deterioration [1,9]. As our data do not clearly demonstrate that spinal decompression modified the underlying course of ALS, it might be still reasonable to consider spinal decompression surgery in selected ALS patients if clinically indicated. However, it is worth noting that although transient improvement in strength may occur following spinal decompression surgery, all ALS patients went on to develop worsening neuromuscular weakness [1,6,8].

The strength of our study includes the large internal database and large referral base as a tertiary medical center. Our study was limited as it was a retrospective chart review. Furthermore, not all patients had EMG prior to spine surgery, so the EMG results may have been influenced by the surgical procedure. Finally, a more accurate approach to evaluate the influence of spinal decompression surgery on ALS progression could be a direct comparison of ALSFRS-R score rate change before and after surgery, which we were unable to analyze due to data limitations.

## Conclusions

The diagnosis of ALS should be considered even in patients presenting with chronic progressive limb weakness with apparent cervical or lumbosacral spine stenosis. Neurologic consultation is helpful, particularly in patients with a lack of prominent pain, ongoing weight loss, presence of bulbar signs, and prominent axonal loss changes in EMG. Spinal decompression surgery could still be considered in selected ALS patients who have prominent radicular pain or when spinal stenosis is the main cause of clinical manifestations. 
